# Low Incidence of Amyloid Related Imaging Abnormalities over 104 Weeks in a Phase 2 Study of Amyloid Anti‐Oligomer Agent ALZ‐801: Phase 2 Study Results in Biomarker Positive APOE4 Carriers with Early Alzheimer’s Disease (AD)

**DOI:** 10.1002/alz.092653

**Published:** 2025-01-09

**Authors:** Patrick Kesslak, Susan Abushakra, Luc Bracoud, John A. Hey, Jerome Barakos, Jakub Hort, Niels D. Prins, Joyce Suhy, Aidan Power, Martin Tolar

**Affiliations:** ^1^ Alzheon Inc., Framingham, MA USA; ^2^ Alzheon, Inc., Framingham, MA USA; ^3^ Clario, Inc., Lyon France; ^4^ Clario, San Mateo, CA USA; ^5^ Memory Clinic, Department of Neurology, Motol University Hospital, Prague Czech Republic; ^6^ Brain Research Center, Amsterdam Netherlands; ^7^ Clario, Inc., San Mateo, CA USA

## Abstract

**Background:**

Oral ALZ‐801 (valiltramiprosate), a brain‐penetrant agent that inhibits amyloid‐oligomer formation is being evaluated in a fully enrolled APOLLOE4 Phase 3 trial in APOE4/4 homozygotes with Early Alzheimer’s disease (AD). ALZ‐801 effects on plasma AD biomarkers were evaluated in a 104‐week Phase 2 study in APOE4‐carriers with CSF+ AD biomarkers. APOE4 is a major risk factor for amyloid‐related imaging abnormalities (ARIA) in AD patients. Incidence of ARIA‐H (microhemorrhages, MH; superficial siderosis, SS) and of ARIA‐E (edema/effusion) was analyzed.

**Method:**

This study enrolled 84 subjects (31 APOE4/4, 53 APOE3/4) MMSE ≥22 and CDR‐G 0.5 or 1.0, with positive amyloid and CSF p‐tau181 ≥60 pg/ml. Subjects received ALZ‐801 at 265 mg BID over 104 weeks, with MRI at baseline, 52 and 104 weeks following a standardized protocol including 2D FLAIR and T2* Gradient Echo. Study allowed subjects with any number of MH, but ARIA‐E and SS lesions >1cm were exclusionary. Data were evaluated centrally by Clario neuroradiologists for eligibility assessment and ARIA safety monitoring. The study’s primary outcome was plasma p‐tau_181_ reduction at 104 weeks.

**Result:**

At baseline, mean age and MMSE were 69 years and 26 respectively, with 52% female and 70% MCI subjects. Of 84 enrolled, 83, 75 and 69 subjects had baseline, 52‐week and 104‐week MRI. Baseline MRIs showed >1 MH in 7/83 (8%), and 6/75 subjects (8%) with post‐treatment MRI developed new MH, all asymptomatic. At baseline, 5 subjects had 1‐4 MH, of whom 2 developed new MH (3‐4); 1 subject had >10 MH and developed 29 new MH. 3 subjects developed de novo MH (1‐3). There was no SS or ARIA‐E. Study primary outcome was achieved with significant p‐tau181 reduction (31%, p = 0.045) and 4% Aβ42 reduction (p = 0.042), with memory stabilization over 2 years (Hey, ADPD 2024).

**Conclusion:**

The Phase 2 APOE4‐carrier population with positive CSF biomarkers showed low incidence of microhemorrhage (8%) over 2 years, all asymptomatic, with no siderosis or ARIA‐E. This 265 mg BID ALZ‐801 dose showed significant target engagement and promising cognitive effects in this study. These data suggest a low risk of ARIA with ALZ‐801 in APOE4 carriers with Early AD.

